# Supplementary material for: Protective Effects of Hippophae rhamnoides L. Phenylpropanoids on Doxorubicin-Induced Cardiotoxicity in Zebrafish
Source: Molecules. 2022 Dec 13;27(24):8858. doi: 10.3390/molecules27248858 (PMC9786120; doi:10.3390/molecules27248858)
Supplement: Supplementary file 1 [file molecules-27-08858-s001.zip › molecules-2057505-supplementary.pdf]

molecules

Supplementary Materials

Protective Effects of Hippophae rhamnoides L. Phenylpropanoids on Doxorubicin-Induced Cardiotoxicity in Zebrafish

Gang Li [1], Ming Chu [1], Yingying Tong [1], Yuexin Liang [1], Shenghui Wang [2], Chengjun Ma [1], Zhenhua Wang [1,*] and Wenna Zhou [2,*]

[1] Center for Mitochondria and Healthy Aging, College of Life Sciences, Yantai University, Yantai 264005, China
[2] Department of Pharmaceutical Engineering, School of Life and Health Sciences, Huzhou College, Huzhou 313000, China

Correspondence: skywzh@ytu.edu.cn (Z.W.); 02638@zjhu.edu.cn (W.Z.);
Tel.: +86-185-0638-2875 (Z.W.); +86-183-6729-0912 (W.Z.)

Table of Contents

Figure S1. ^{1}H NMR Spectrum (400 MHz) of p-coumaric acid (P1) (in DMSO-d_6) 2
Figure S2. ^{13}C NMR Spectrum (100 MHz) of p-coumaric acid (P1) (in DMSO-d_6)................3
Figure S3. Structure of p-coumaric acid (P1). 4
Figure S4. ^{1}H NMR Spectrum (400 MHz) of chlorogenic acid (P2) (in DMSO-d_6) 5
Figure S5. ^{13}C NMR Spectrum (100 MHz) of chlorogenic acid (P2) (in DMSO-d_6) 6
Figure S6. Structure of chlorogenic acid (P2)...7
Figure S7. ^{1}H NMR Spectrum (400 MHz) of caffeic acid (P3) (in DMSO-d_6) 8
Figure S8. ^{13}C NMR Spectrum (100 MHz) of caffeic acid (P3) (in DMSO-d_6) 9
Figure S9. Structure of caffeic acid (P3)..10
Figure S10. ^{1}H NMR Spectrum (400 MHz) of ferulic acid (P4) (in DMSO-d_6)....................11
Figure S11. ^{13}C NMR Spectrum (100 MHz) of ferulic acid (P4) (in DMSO-d_6)...................12
Figure S12. Structure of ferulic acid (P4) 13

Figure S1. ^{1}H NMR Spectrum (400 MHz) of p-coumaric acid (P1) (in DMSO-d_6)

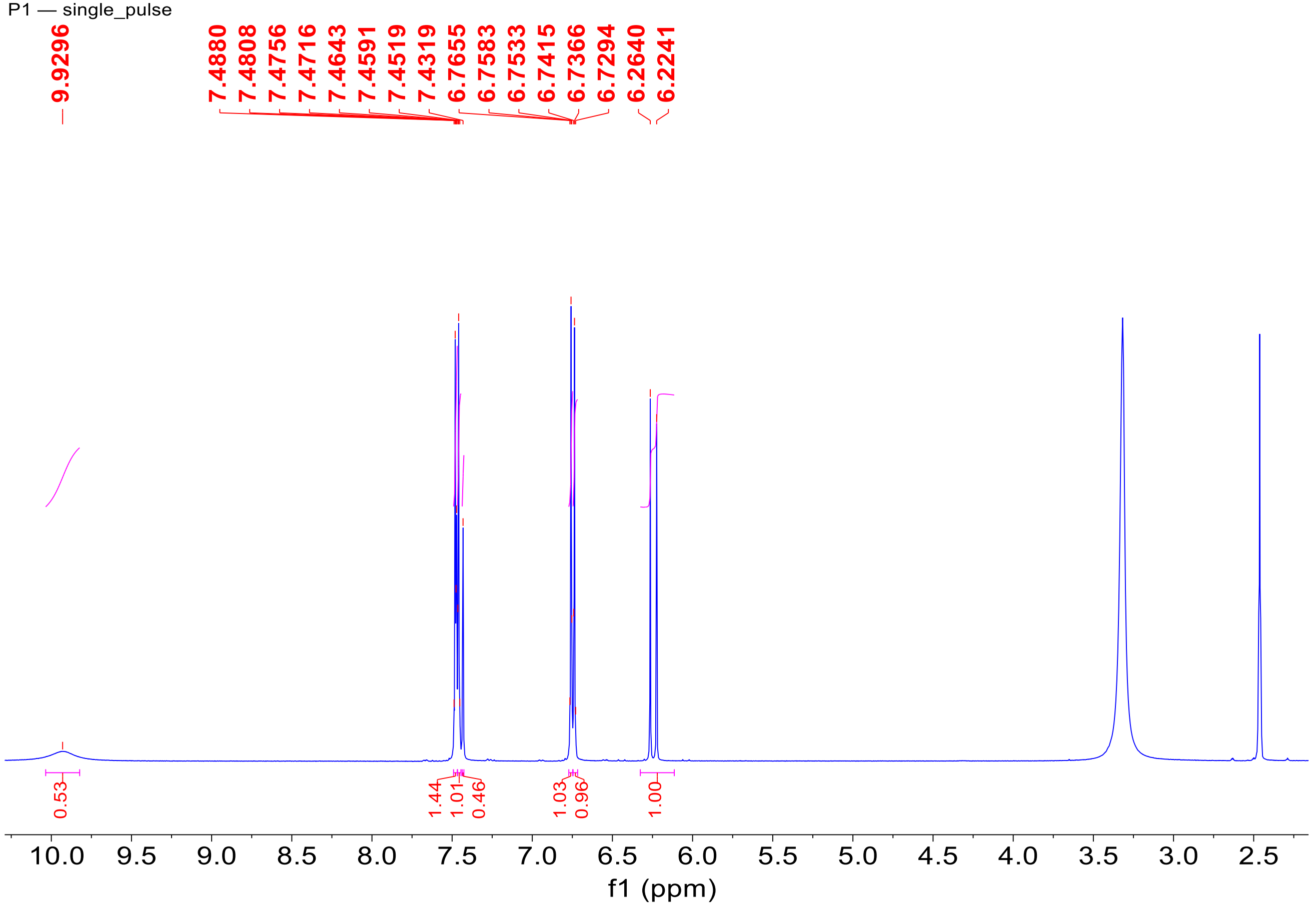

Figure S2. ^{13}C NMR Spectrum (100 MHz) of p-coumaric acid (P1) (in DMSO-d_6)

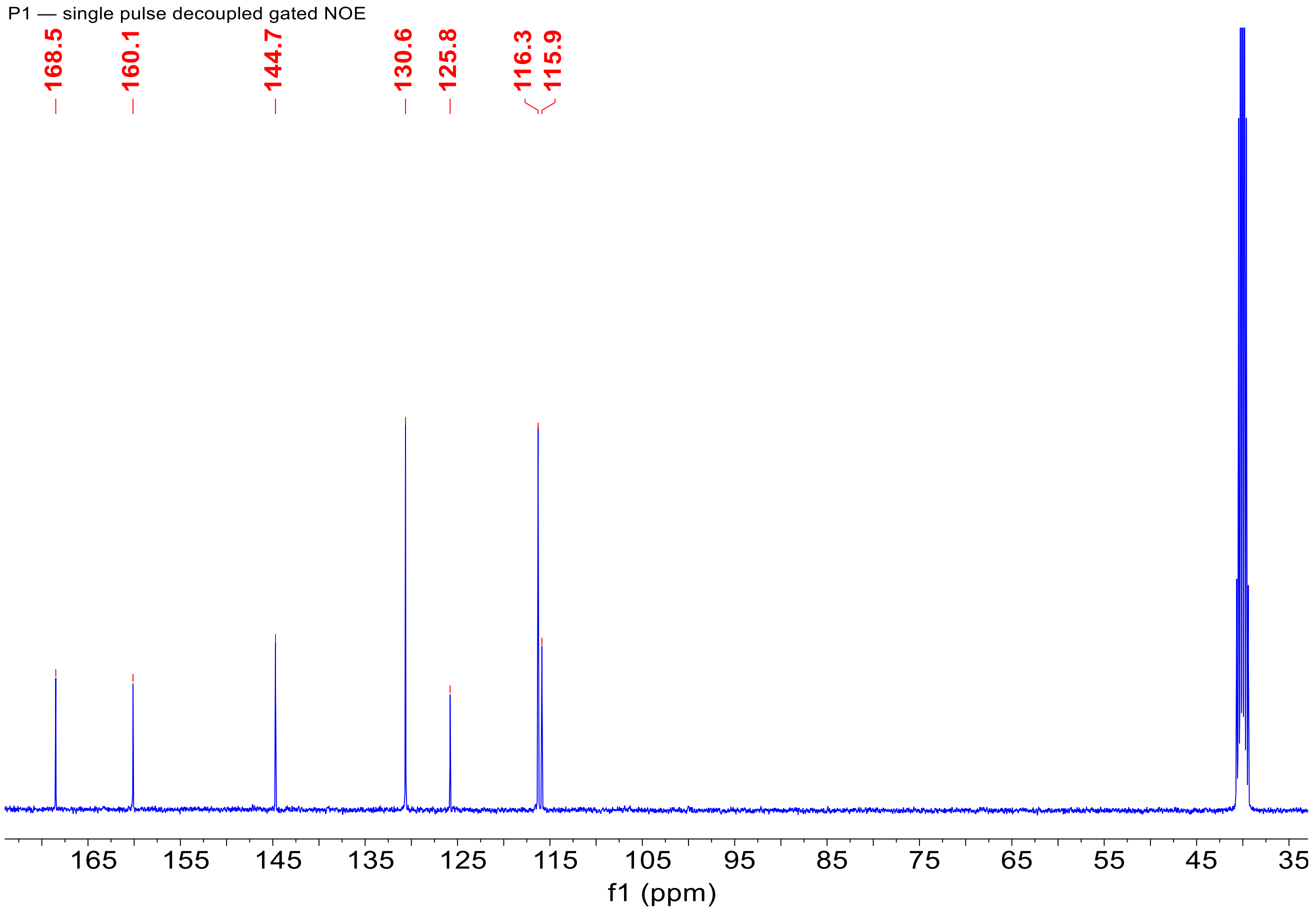

Figure S3. Structure of p-coumaric acid (P1).

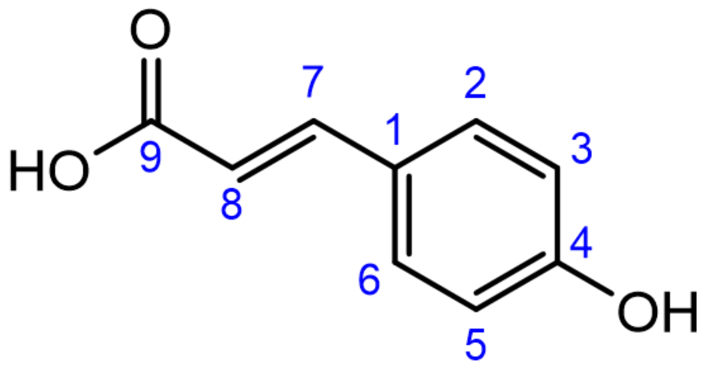

^{1}H-NMR (400 MHz, DMSO-d_6) δ: 9.93 (1H, s, H-4-OH),7.48 (1H, dd, J = 3.7 Hz, H-2), 7.46 (1H, dd, J = 3.7 Hz, H-6),7.43 (1H, ddd, J = 15.9, 3.7, 3.7 Hz, H-7), 6.76 (d, J = 2.0 Hz, 1H, H-3), 6.74 (d, J = 2.0 Hz, 1H, H-5), 6.24 (1H, d, J = 15.9 Hz, H-8); ^{13}C-NMR (100 MHz, DMSO-d_6) δ: 168.5 (C-9), 160.1 (C-4), 144.7 (C-7), 130.6 (C-2, 6), 125.8 (C-1), 116.3 (C-8), 115.9 (C-3, 5). The data correspond to p-coumaric acid.

Figure S4. ^{1}**H NMR Spectrum (400 MHz) of chlorogenic acid (P2) (in DMSO-d_6)**

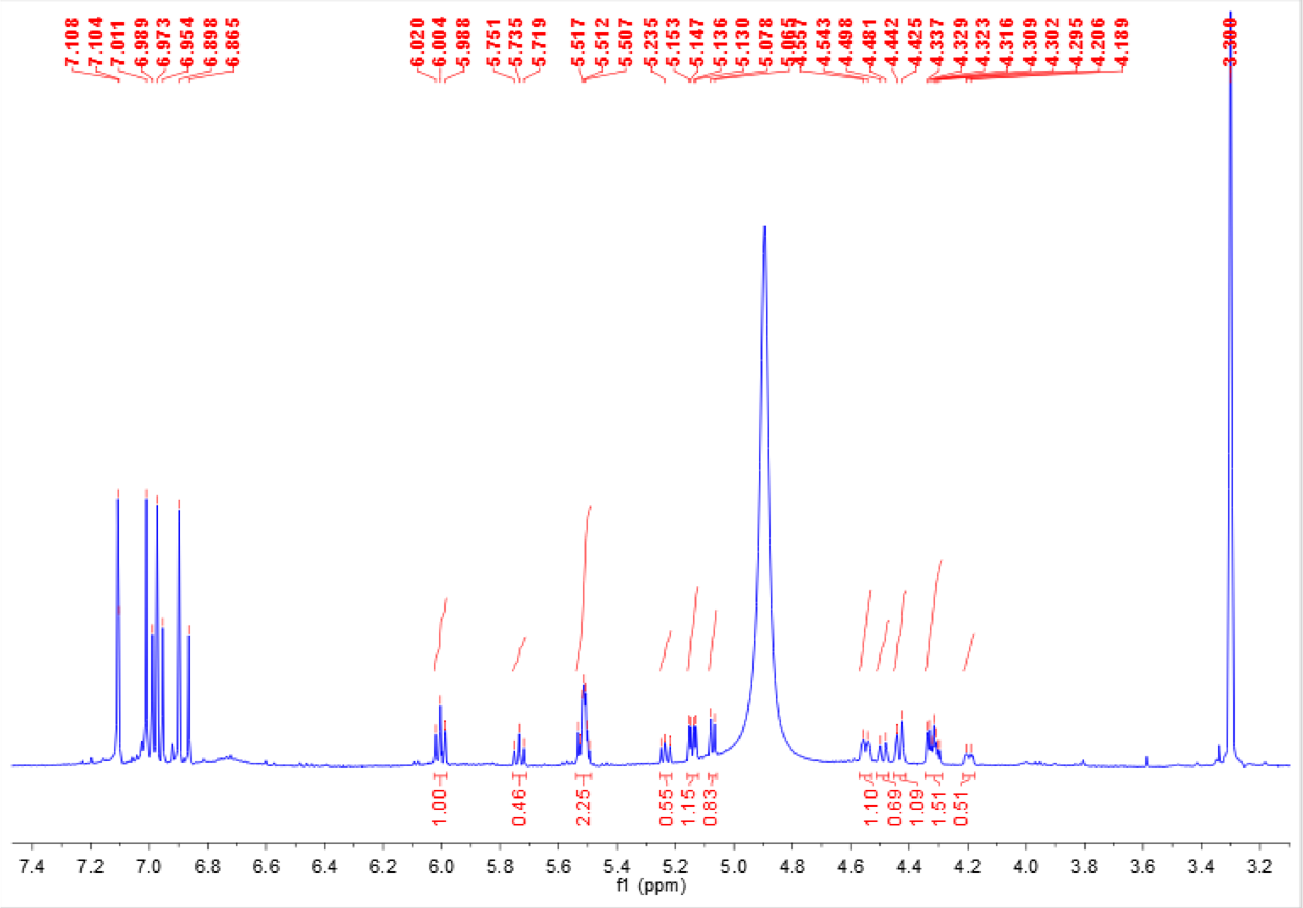

Figure S5. ^{13}C NMR Spectrum (100 MHz) of chlorogenic acid (P2) (in DMSO-d_6)

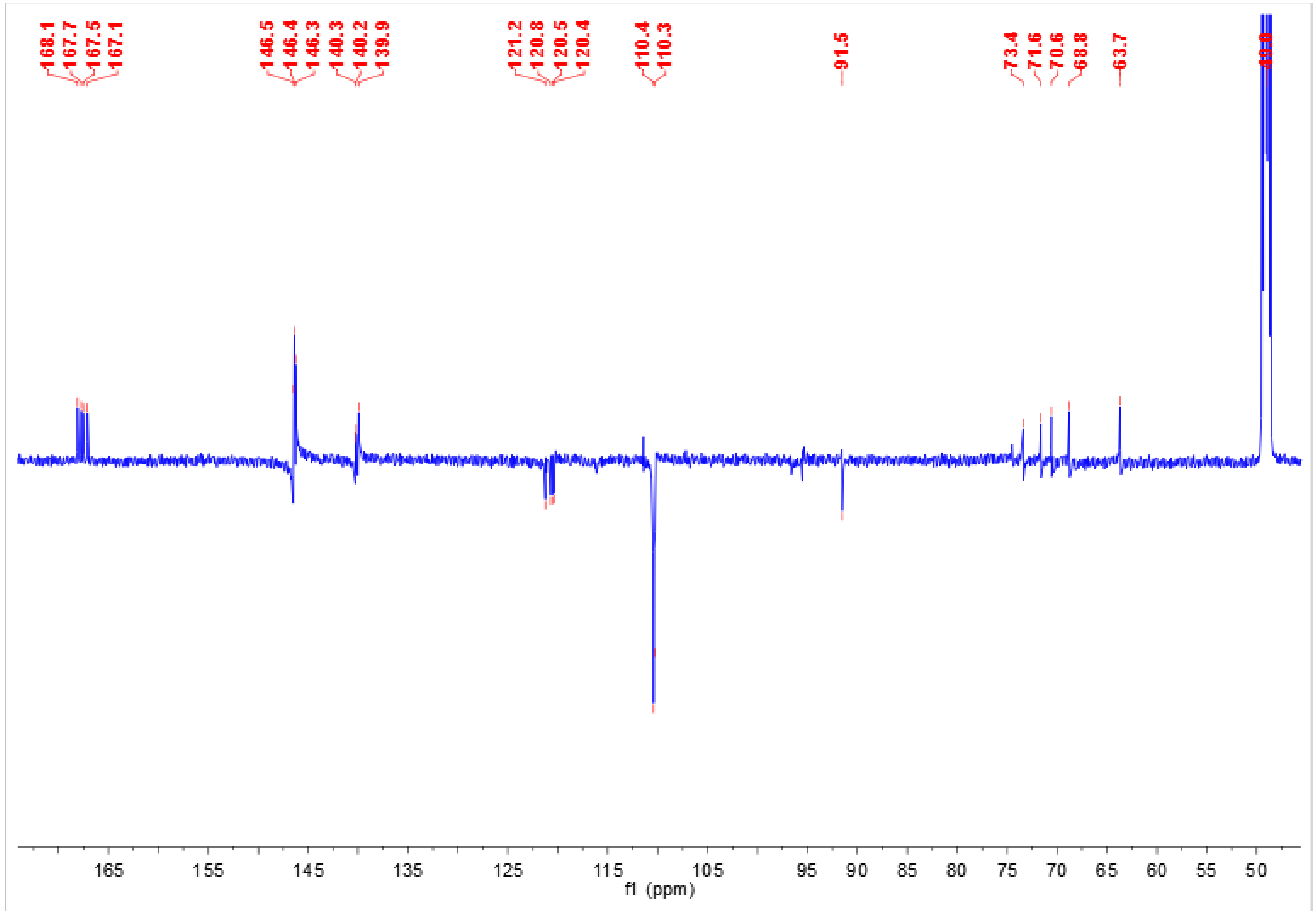

Figure S6. Structure of chlorogenic acid (P2).

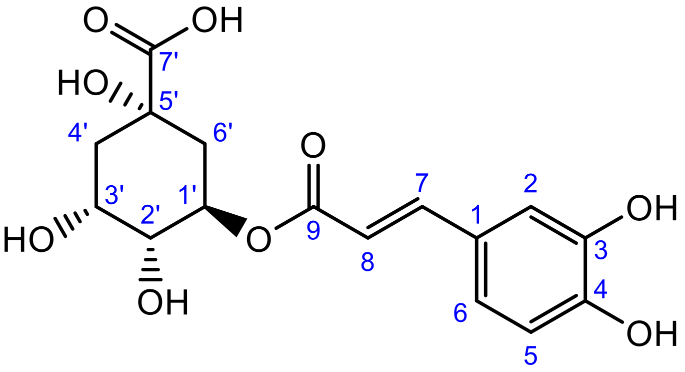

^{1}H-NMR (400 MHz, DMSO-d_6) δ: 7.43 (1H, d, J = 16 Hz, H-7), 7.06 (1H, d, J = 1.6 Hz, H-2), 6.96 (1H, dd, J = 8.0, 1.6 Hz, H-6), 6.75 (1H, d, J = 8.0 Hz, H-5), 6.22 (1H, d, J = 16.0 Hz, H-8), 5.15 (1H, dt, J = 10.4, 4.8 Hz, H-1′), 3.88 (1H, d, J = 3.2 Hz, H-2′), 3.46 (1H, dd, J = 9.6, 2.4 Hz, H-3′), 1.60~1.98 (4H, m, H-4′, 6′); ^{13}C-NMR (100 MHz, DMSO-d_6) δ: 176.5 (C-7′), 166.4 (C-9), 148.5 (C-4), 145.8 (C-3), 144.8 (C-7), 125.5 (C-1), 121.3 (C-6), 115.9 (C-5), 114.8 (C-8), 114.6 (C-2), 75.2 (C-1′), 73.2 (C-2′), 71.6 (C-4′), 71.4 (C-6′), 39.5 (C-5′), 38.1 (C-3′). The data correspond to chlorogenic acid.

Figure S7. ^{1}**H NMR Spectrum (400 MHz) of caffeic acid (P3) (in DMSO-*d*$_6$)**

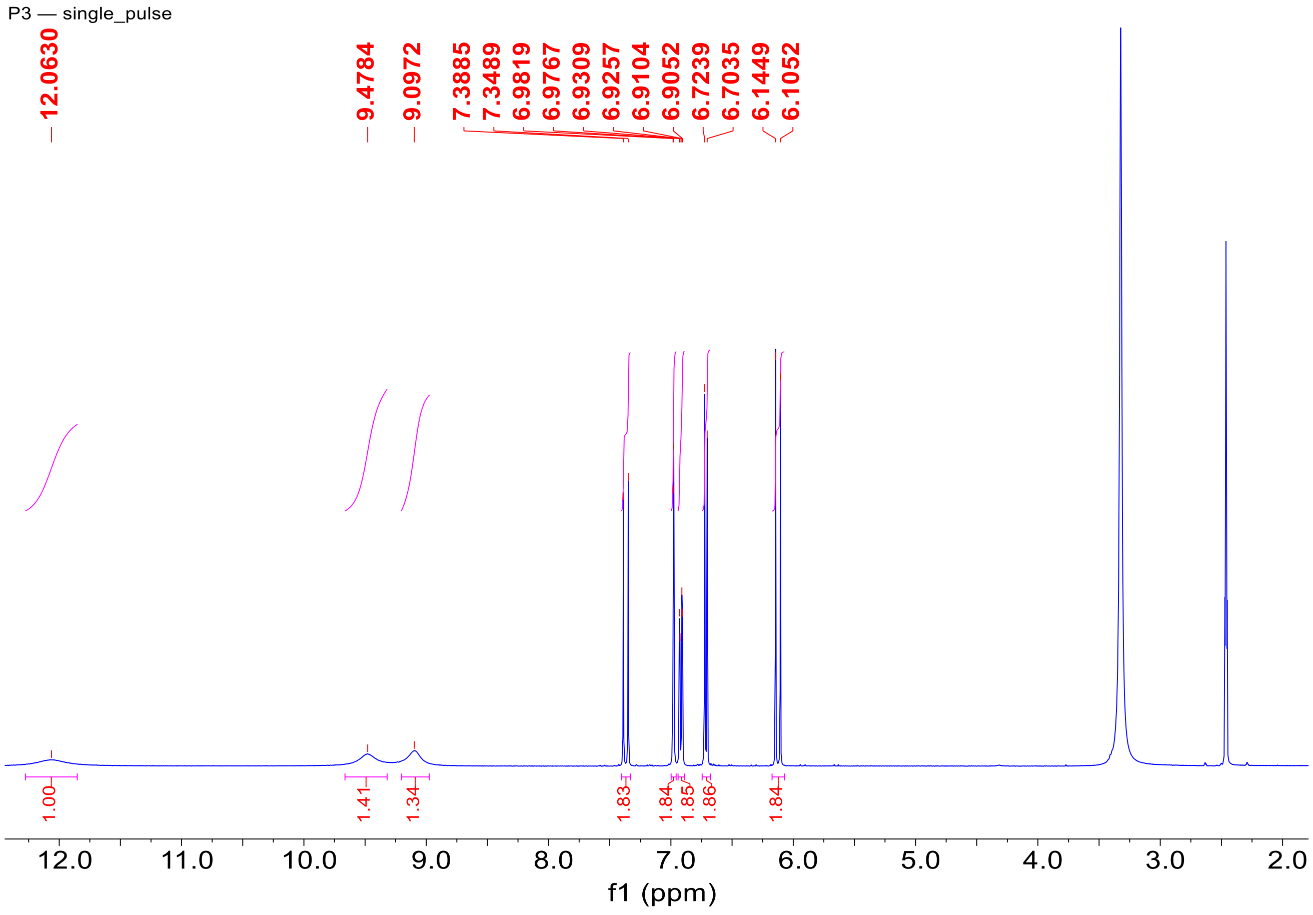

Figure S8. ^{13}C NMR Spectrum (100 MHz) of caffeic acid (P3) (in DMSO-d_6)

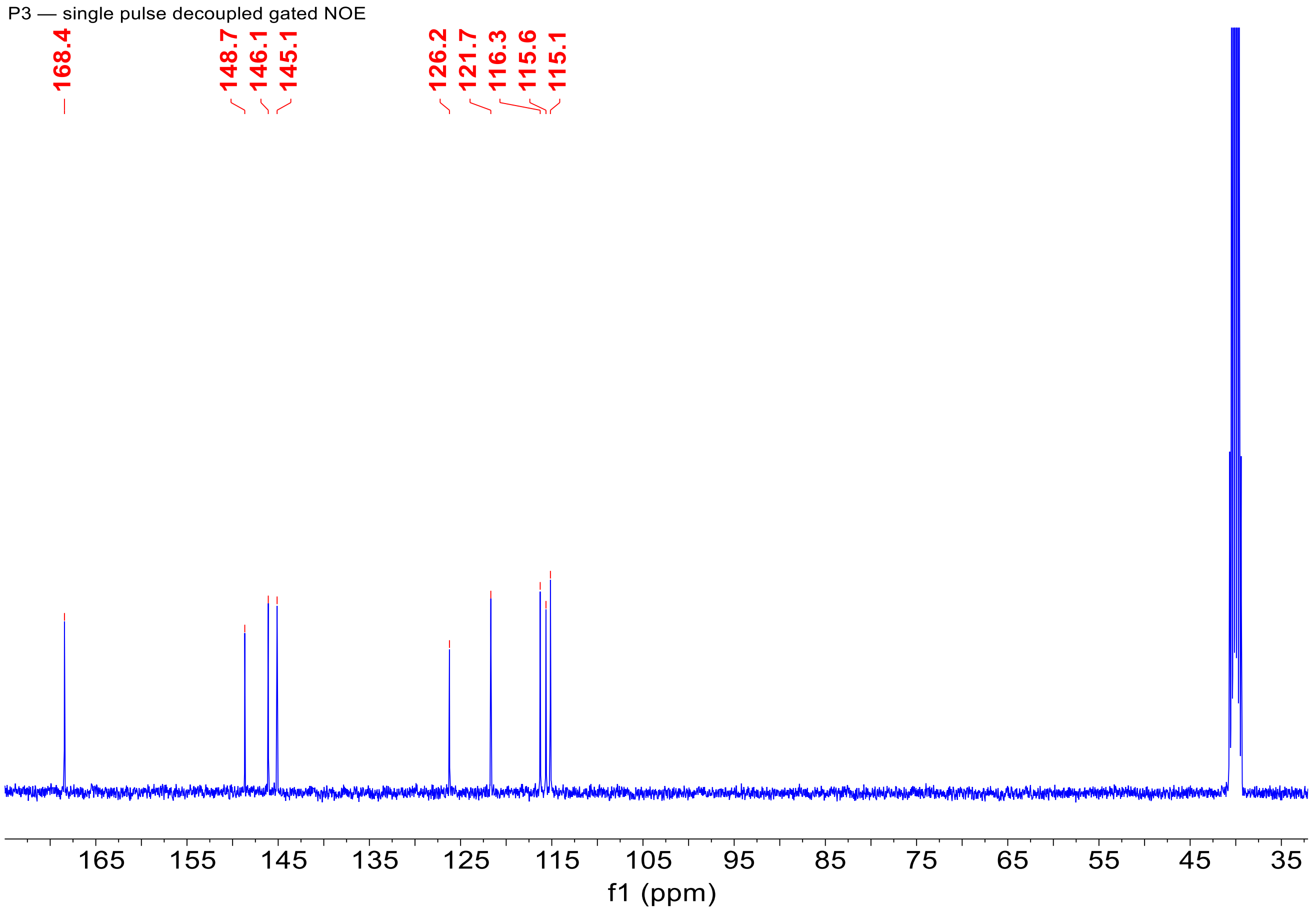

Figure S9. Structure of caffeic acid (P3)

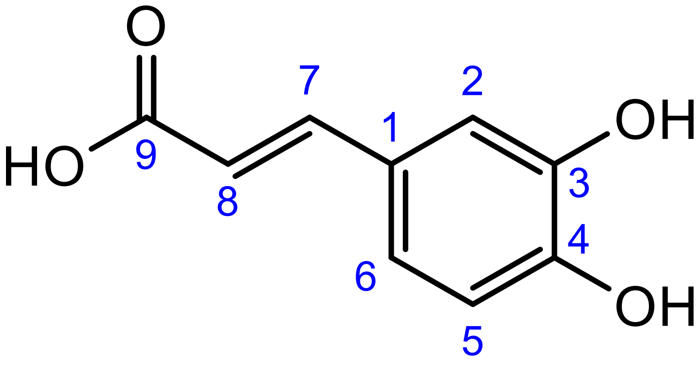

^{1}H-NMR (400 MHz, DMSO-d_6) δ: 12.05 (1H, s, H-9-OH), 9.48 (1H, s, H-3-OH), 9.10 (1H, s, H-4-OH), 7.37 (1H, d, J = 15.9 Hz, H-7), 6.98 (1H, d, J = 2.1 Hz, H-2), 6.92 (1H, dd, J = 8.2, 2.1 Hz, H-6), 6.71 (1H, d, J = 8.2 Hz, H-5), 6.13 (1H, d, J = 15.9 Hz, H-8); ^{13}C-NMR (100 MHz, DMSO-d_6) δ: 168.4 (C-9), 148.7 (C-7), 146.1 (C-4), 145.1 (C-3), 126.2 (C-1), 121.7 (C-6), 116.3 (C-5), 115.6 (C-8), 115.1 (C-2). The data correspond to caffeic acid.

Figure S10. ^{1}H NMR Spectrum (400 MHz) of ferulic acid (P4) (in DMSO-d_6)

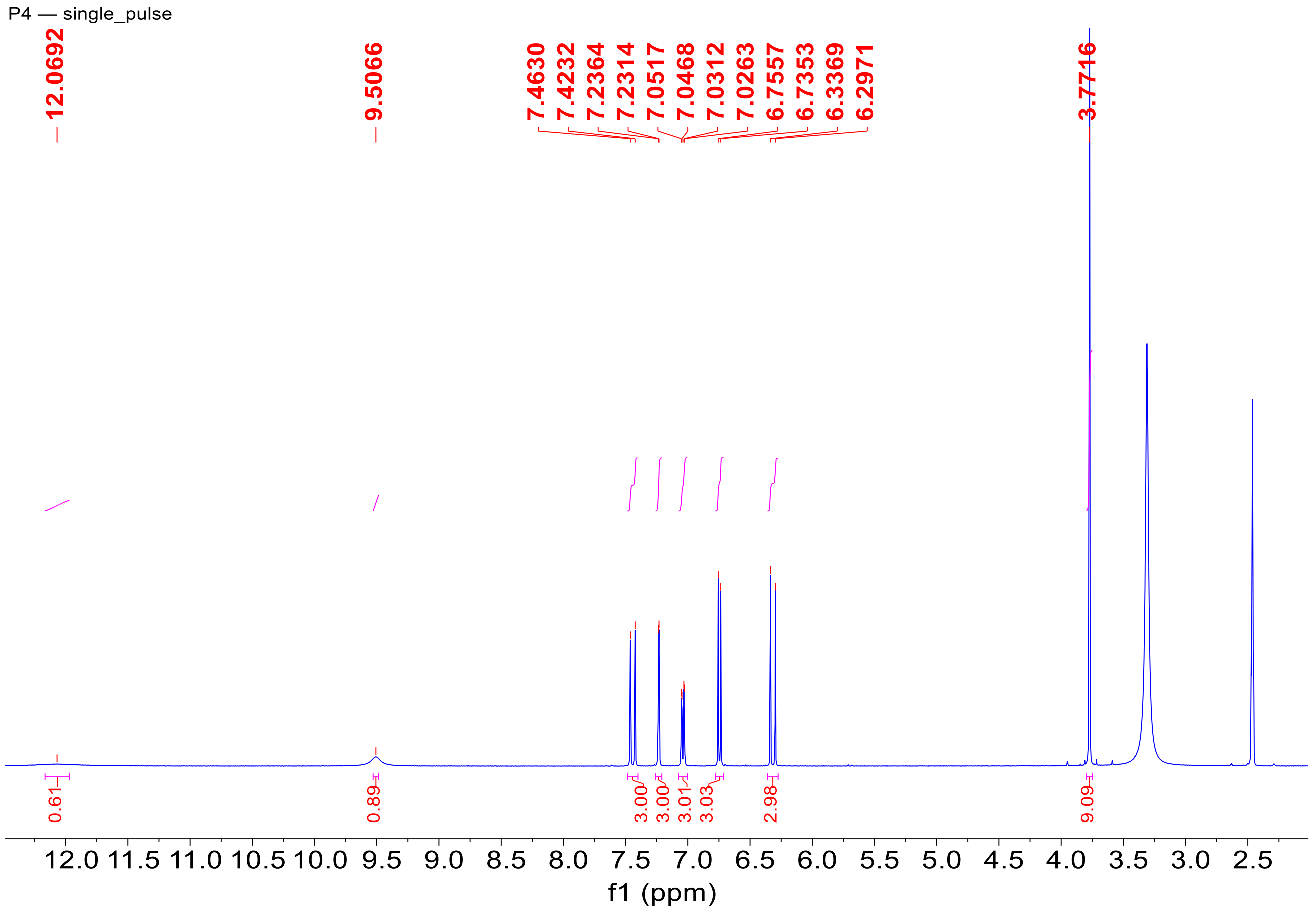

Figure S11. ^{13}**C NMR Spectrum (100 MHz) of ferulic acid (P4) (in DMSO-*d*$_6$)**

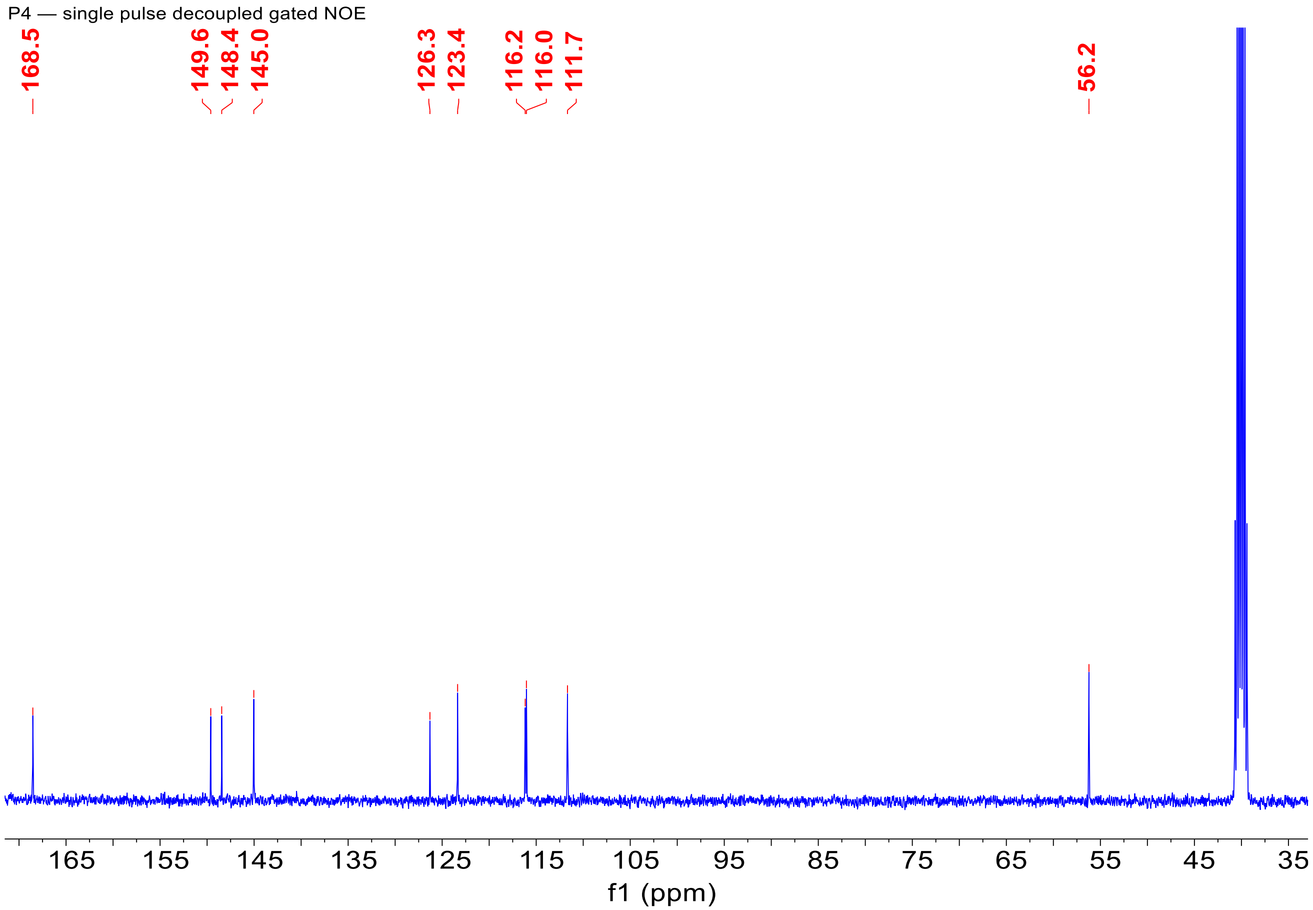

Figure S12. Structure of ferulic acid (P4)

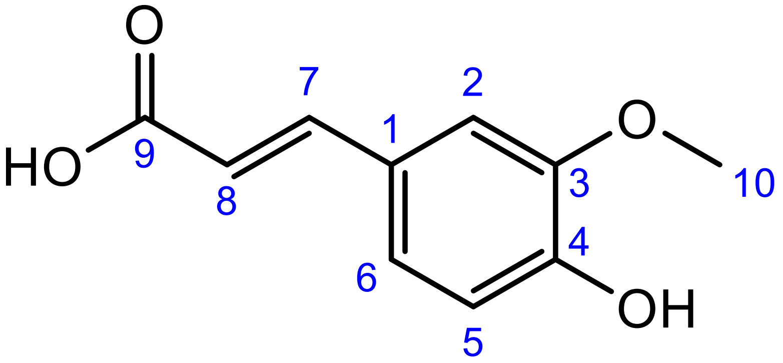

^{1}H-NMR (400 MHz, DMSO-d_6) δ: 12.07 (1H, s, H-9-OH), 9.51 (1H, s, H-4-OH), 7.44 (1H, d, J = 15.9 Hz, H-7), 7.23 (1H, d, J = 2.0 Hz, H-2), 7.04 (1H, dd, J = 8.2, 2.0 Hz, H-6), 6.75 (1H, d, J = 8.1 Hz, H-5), 6.32 (1H, d, J = 15.9 Hz, H-8), 3.77 (3H, s, H-10);
^{13}C-NMR (100 MHz, DMSO-d_6) δ: 168.5 (C-9), 149.6 (C-3), 148.4 (C-4), 145.0 (C-7), 126.3 (C-1), 123.4 (C-6), 116.2 (C-5), 116.0 (C-8), 111.7 (C-2), 56.2 (C-10). The data correspond to ferulic acid.
